# Arrhythmic Burden and Outcomes in Pulmonary Arterial Hypertension

**DOI:** 10.3389/fmed.2019.00169

**Published:** 2019-07-23

**Authors:** Jennifer T. Middleton, Angshuman Maulik, Robert Lewis, David G. Kiely, Mark Toshner, Athanasios Charalampopoulos, Andreas Kyriacou, Alexander Rothman

**Affiliations:** ^1^Sheffield Pulmonary Vascular Disease Unit, Royal Hallamshire Hospital, Sheffield Teaching Hospitals NHS Foundation Trust, Sheffield, United Kingdom; ^2^Department of Infection, Immunity and Cardiovascular Disease, Medical School, University of Sheffield, Sheffield, United Kingdom; ^3^Department of Cardiology, Northern General Hospital, Sheffield Teaching Hospitals NHS Foundation Trust, Sheffield, United Kingdom; ^4^Department of Medicine, Addenbrooke's Hospital, University of Cambridge, Cambridge, United Kingdom; ^5^Royal Papworth Hospital NHS Foundation Trust, Cambridgeshire, United Kingdom

**Keywords:** arrhythmia, pulmonary arterial hypertension, right heart failure, atrial fibrillation, atrial flutter, ventricular tachycardia

## Abstract

Pulmonary arterial hypertension (PAH) is a devastating, life-limiting disease driven by small vessel vascular remodeling leading to a rise in pulmonary vascular resistance (PVR). Patients present with a range of symptoms including shortness of breath, exercise intolerance, palpitations or syncope. Symptoms may be related to vascular disease progression or arrhythmia secondary to the adaptation of the right heart to pressure overload. Arrhythmic burden is high in patients with left heart disease and guideline-based treatment of arrhythmias improves quality of life and prognosis. In PAH the incidence and prevalence of arrhythmias is less well-defined and there are no PAH-specific guidelines for arrhythmia management. We undertook a literature search identifying 13 relevant papers; detection of arrhythmias was acquired from 12-lead electrocardiogram (ECG) or Holter monitors. In all forms of pulmonary hypertension (PH) the prevalence of supraventricular arrhythmias (SVA) was 26–31%, ventricular arrhythmias (VA) 24% and a 5-year incidence of SVA ~13.2–25.1%. Prevalence and incidence of arrhythmias in PAH is less clear due to limited study numbers and the heterogenous nature of the patient population studied. For arrhythmia treatment, only single-arm studies of therapeutic strategies were reported using antiarrhythmic drugs (AAD), direct current cardioversion (DCCV) and ablation. Periods between ECG or Holter have not been investigated, highlighting the possibility that significant arrhythmias may be undetected. Advances in monitoring allow long-term surveillance via implanted/non-invasive monitors. Use of such technologies may provide an accurate estimate of incidence and prevalence of arrhythmias in patients with PAH, further defining relationships to adverse outcomes, and therapeutic options.

## Introduction

The recent World Symposium classifies pulmonary hypertension into five groups based on underlying cause (1). Group one incorporates pulmonary arterial hypertension including familial and idiopathic causes; group two is secondary to left sided heart disease; group three to lung disease and hypoxia; group four is secondary to chronic thromboembolic disease (CTEPH) and group five includes miscellaneous causes including sarcoidosis and hematological disease (1). Increased right ventricular pressure and volume overload of the right heart leads to structural changes that impair left ventricular filling (2) and function which may predispose to the development of arrhythmias. In this review we will discuss the prevalence and incidence of arrhythmia in PAH and the potential treatment options.

## Cardiac Arrhythmias: Classification and Mechanism

**Figure 1:**
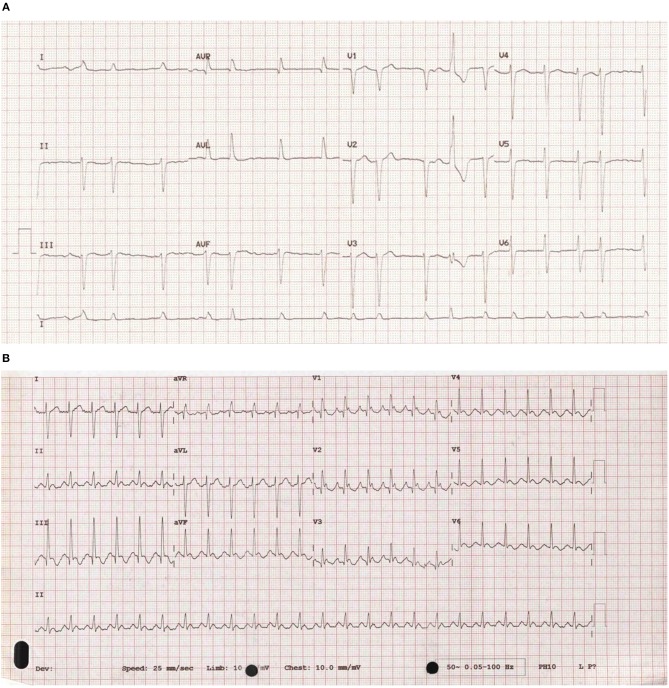
**(A)** An ECG of atrial fibrillation. An example of an ECG taken from a patient with atrial fibrillation showing uncoordinated atrial activity resulting in an irregular ventricular rate. **(B)** An ECG of atrial flutter. An example of an ECG taken from a patient with atrial flutter. It is characterized by rapid, regular atrial depolarizations (typically, but not always at a rate of 300 bpm) resulting in a ventricular rate of 150 bpm.

**Figure 2:**
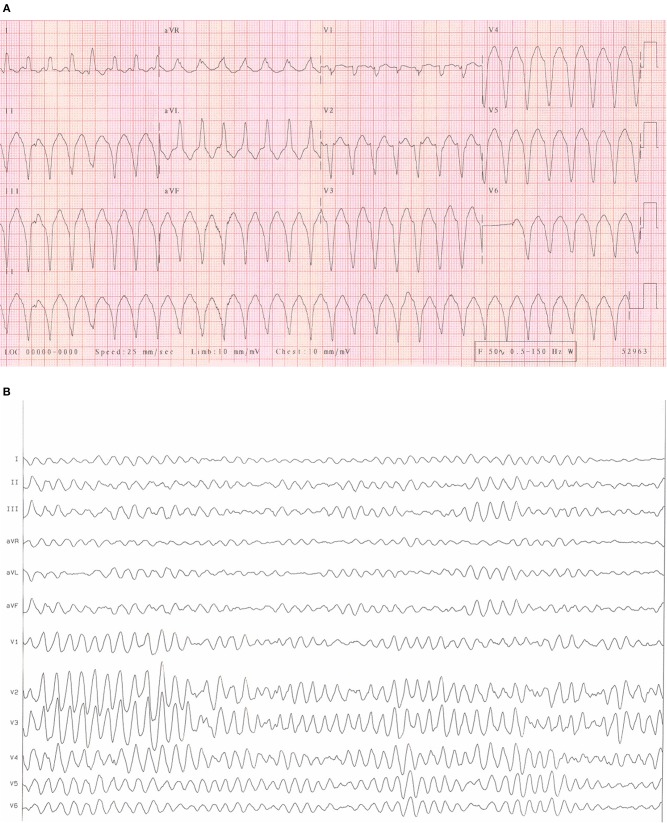
**(A)** An ECG of ventricular tachycardia. An example of an ECG from a patient with sustained monomorphic ventricular tachycardia. It is characterized by regular, broad QRS complexes of similar morphology often over 100 bpm. **(B)** An ECG of Ventricular fibrillation. An example of an ECG from a patient with ventricular fibrillation. It is characterized by rapid, erratic ventricular activity as the heart fibrillates rather than pumps effectively. This rhythm quickly degenerates into cardiac arrest.

Cardiac arrhythmias are caused by either disruption of the cardiac action potential or structural/functional changes to the heart that result in abnormalities of electrical conduction. Classification is made based on heart rate (bradycardic or tachycardic) and sub-divided based on site of origin (supraventricular or ventricular arrhythmia). In the left and right atrium, dilatation and stretch results in structural, and ion channel adaptations resulting in an increased development of SVA's including atrial fibrillation (AF) and atrial flutter (see Figures 1A,B). Subsequent loss of atrial contraction leads to underfilling of the ventricle and reduction in cardiac output (CO). VA's can be precipitated by a dilated, dysfunctional ventricle, or altered ventricular substrate in the form of scar (3), giving rise to pro-arrhythmic myocardium (see Figures 2A,B). In left sided heart failure there is an increased risk of arrhythmia, which when present often exacerbates left ventricular failure (3, 4). Impaired left ventricular function results in reduced CO due to inadequate filling and contraction leading to compensatory hypertrophy and dilatation of the left ventricle. These changes increase sarcomere length and alter sensitivity to calcium leading to more forceful ventricular contraction (5), the resultant ion channel remodeling (6) can make myocardium pro-arrhythmic (3). Bradyarrhythmia presents often secondary to changes in heart structure, nodal fibrosis, or rate-limiting medication used to treat heart failure.

Current guidelines for the treatment of arrhythmia specifically relate to patients with left sided heart disease (4–9). Management strategies include AAD's that alter the action of specific ion channels to stabilize myocardium; anticoagulation to reduce stroke risk in AF (6); synchronized DCCV to revert to normal sinus rhythm (NSR); electrophysiology studies (EPS); and/or ablation to identify abnormal heart rhythms using intracardiac catheters and ablation to disrupt abnormal conduction pathways and finally permanent pacemakers or internal defibrillator devices (7, 10). Guidance for the treatment of arrhythmias in patients with PAH is lacking despite the potential harmful/unknown effects of AAD's and or therapies in this cohort (7).

### Arrhythmias in PAH

Patients with PAH often describe palpitations and/or syncope. The incidence and prevalence of cardiac arrhythmias in PAH is not well-described, however a number of recent studies describe a significant arrhythmic burden in all forms of PH, and a relationship to clinical outcome. These studies are based on “snapshots” in time using 12 lead ECGs or short-term Holter monitors. We undertook a literature-based review searching PubMed with the search terms “pulmonary arterial hypertension, supraventricular, and ventricular arrhythmias, atrial fibrillation/flutter, ventricular tachycardia, and mode of death,” 13 relevant papers were reviewed as well as PAH registry data. A review of the REVEAL PAH registry data demonstrated a relationship between increased heart rate and adverse outcomes (11, 12) and during follow up, Burger et al. found 5.1% of patients were admitted secondary to an arrhythmia, however further details were not provided (13).

#### Supraventricular Arrhythmias

##### Prevalence

In a multi-center, observational study (14), patients with PAH/inoperable CTEPH attending routine outpatients had their notes reviewed retrospectively for evidence of AF or atrial flutter. 297 patients were identified as fulfilling criteria (PAH 266/CTEPH 31) with 90% on PAH specific medication. An ECG diagnosis of AF or atrial flutter was noted in 79 (26.5%), approximately 50% of those were deemed to be paroxysmal in nature. Patients with AF were as a group, of increased age, male gender with a higher systolic blood pressure (BP), a reduced left ventricular ejection fraction (LVEF), and had a higher rate of chronic obstructive lung disease (COPD). At right heart catheterization (RHC), the AF group had a higher pulmonary capillary wedge pressure (PCWP), and lower pulmonary vascular resistance, potentially suggesting a more elderly population with a greater burden of co-morbid condition. There was no difference found in mean pulmonary artery pressure (PAP) or CO. Results suggest that a diagnosis of permanent or paroxysmal AF/atrial flutter in PAH confers an increased mortality. The use of 12-lead ECGs alone and the unbalanced co-morbidities of patients with and without AF/atrial flutter masked the true burden of arrhythmias in patients with PAH (14).

Rottlaender et al. (15), investigated patients with all forms of PH who developed SVA in a retrospective study of 225 patients. Thirty one percentage of patients recruited were found to have AF (41% paroxysmal) and it was apparent that AF was more likely if secondary to PH-left heart disease and less common in CTEPH. All patients identified as having AF deteriorated clinically at the time of arrhythmia. The true prevalence of SVA was again difficult to determine due to the inclusion of all types of PH. In contrast Ruiz-Cano et al. retrospectively reviewed 282 patients with PAH. In total 28 SVA episodes were detected: AF 12 patients (42.8%); atrial flutter 12 (42.8%); other SVA 4 (14.2%), 82% of SVA episodes resulted in symptoms and clinical deterioration / worsening right heart failure (RHF). Time from PAH diagnosis to SVA was approximately 60 months and time from SVA diagnosis to death/transplant was 17.8 months suggesting increased morbidity and mortality with the development of SVA.

##### Incidence

A number of studies investigated incidence through prospective/retrospective cohort studies (summarized in Table 1), with 6-year incidence of SVA in PAH approximately 15.8% and in all forms of PH a 5-year incidence of between 13.2 and 25.1% (17–21). In a retrospective, cohort study, Cannillo et al. examined patients with PAH, lung disease-associated PH, and CTEPH who had NSR at baseline on a 12-lead ECG, patients with previous SVA diagnosis were excluded (18). Retrospective analysis of ECG's was undertaken during follow-up, if admitted or if they developed palpitations. Holter monitors were only fitted in patients reporting palpitations, not as a screen for asymptomatic SVA's. 77 patients met criteria, 17 patients (22%) were newly diagnosed with SVA during the follow-up period (35 months). Patients were found to have persistent AF in 8 patients (47%), permanent AF 3 patients (17%), paroxysmal SVA 3 patients (17%), atrial ectopic tachycardia 2 patients (12%), right atrial flutter 2 patients (12%) and paroxysmal AF (PAF) in 1 patient (6%), other SVA 1 patient (6%). Diagnosis of SVA's occurred on average 15.1 months after PH diagnosis and was associated with worsening parameters (World health organization (WHO)-functional class/6-min walk test (6MWT)/brain natriuretic protein (BNP), and increased mortality. This suggests SVA to be a manifestation of more severe PAH [as per Tongers (21) and Ruiz-Cano (16)]. Severe PAH resulted in more hospital admissions and closer monitoring by default, potentially introducing detection bias of SVA in these patients. Consistent with these findings, Mercurio et al. (17) again looked at PAH only and specifically idiopathic PAH and systemic sclerosis-associated pulmonary hypertension (SSc-PH). A prospective, single center study recruited 317 patients in total (116 idiopathic PAH) and of these 42 developed SVA. In keeping with Smith et al. (14) these patients had higher baseline PCWP and in this case right atrial pressure (RAP). In 90.1% cases the onset of SVA resulted in clinical worsening and RHF. A 5-year incidence of SVA in idiopathic PAH and SSc-PAH was found to be 13.2%, lower than previously documented by Olsson et al. in a similar PH group (20). Olsson et al. described a 5-year incidence of 25.1% of SVA in PAH and inoperable CTEPH. Patients again had NSR at baseline and diagnosis of arrhythmia was based on 12-lead ECG's only. Forty-Eight patients (20%) had at least one episode of SVA. They also showed patients with persistent AF to have a worse prognosis than those with PAF or NSR (20). This followed on from a previous study by Tongers et al. (21), that retrospectively recruited 231 patients under follow-up for PAH and inoperable CTEPH. Thirty-one episodes of SVA were noted on 12-lead ECG in 27 patients (Atrial flutter 15, AF 13, and other SVA 3). Episodes were again associated with marked clinical deterioration and RHF (84% SVA episodes), improving if NSR was restored. Interestingly this study appears to show an increased mortality in AF patients where NSR could not be restored. All 15 patients with atrial flutter had NSR, restored with medication (1), DCCV (6), overdrive pacing (15) or ablation (5), with one death during follow-up. In the AF group only 2 patients had NSR restored by DCCV. NSR in the remaining 9 was not achieved despite multiple medications and DCCV, with 8 deaths during follow-up. This suggests an increased mortality in AF and PAH if NSR is not achieved. It would also appear that treatment measures in the AF group were less aggressive (ablation was not attempted) but the study was from 2007 so potentially now outdated as ablation is more common place today.

**Table 1:** Summary of literature reviewing SVA in PH.

**Reference**	**Type of study**	**Pathology**	**Number of patients**	**Demographics in arrhythmia group**	**Prevalence/ incidence**	**Investigation**	**Rhythm identified**	**NSR restored?**	**Treatment given**	**Outcome**
Smith et al. (14)	Retrospective 4-year study	PAH CTEPH	PAH 266 CTEPH 31	F:M 53:14% Age 61.8 ± 14 MeanPAP 44 ± 11	Prevalence	12-Lead ECG	AF/atrial flutter 50% paroxysmal	31 patients	86% AAD's 14% Not specified	
Rottlaender et al. (15)	Retrospective 4-year study	All types of PH	Total 225	F:M 36:64% Age 71.2 ± 1.1 MeanPAP 40.8 ± 1.6	Prevalence	12 Lead ECG	AF 41% paroxysmal	Not discussed	Not discussed	Permanent AF = Clinical deterioration.
Ruiz- Cano et al. (16)	Retrospective, 4-year, single center study	PAH	Total 282	F:M 61:39% Age 47.3 ± 4.3	Prevalence	Medical notes/12-lead ECG	AF/atrial flutter /atrioventricular node re-entry tachycardia (AVNRT)	Attempted in all	All underwent EPS +/− ablation	Restoration of NSR = clinical improvement 4 SVA recurrence
Mercurio et al. (17)	Prospective, single center study	Idiopathic PAH PAH-SSc	116 IPAH 201 SSc-PH	F:M 71-29% Age 59+-12.1 MeanPAP 47.3+-14.3	Incidence at 5 years 13.2%		AF/atrial flutter/atrial tachycardia	Attempted in all	90.1% SVA = clinical worsening/RHF.	Restoration of NSR = clinical improvement
Cannillo et al. (18)	Retrospective, single center study? type of PH experienced SVA	PAH PH secondary to lung disease CTEPH (inoperable)	Total 77	F:M 55:45% Mean age 63 MeanPAP 43	Incidence	12 Lead ECG Holter if symptomatic	AF/atrial flutter 4 paroxysmal (23%).	13 patients	AAD's/DCCV/ablation SVA = worsening prognostic parameters and RHF. NSR restored in 11 cases	Recurrent SVA in 9 patients
Li-Wen et al. (19)	Prospective, cohort 6-year study	Idiopathic PAH (all taking PH meds)	Total 280	F:M 72.5:27.5% Age 39+-15 MeanPAP 64+-18	Incidence at 6 years (15.8%)		AF/atrial flutter /atrial tachycardia	21 patients	AAD's/DCCV/ablation SVA = clinical deterioration	NSR restored = clinical improvement 6 x recurrence
Olsson et al. (20)	Prospective 5-year, single center study	PAH or CTEPH (inoperable) NSR at baseline, all receiving PH meds	157 PAH 82 Inoperable CTEPH	F:M 65:35% Age 58+-9 MeanPAP 52+-8	Incidence at 5 years (25.1%)	12-Lead ECG	AF/atrial flutter	21/24 atrial flutter 16/24 AF	AAD's/overdrive pacing /DCCV/ablation	NSR = clinical improvement
Tongers et al. (21)	Retrospective 6-year study	PAH CTEPH (inoperable)	Total 231	F:M 65:35% Age 49+-13 MeanPAP 50+-10	Cumulative incidence of 11.7% and annual risk of 2.8%/ patient.	12 Lead ECG AVNRT diagnosed on EPS	AF/atrial flutter /AVNRT 4 SVA recurrence	15/15 atrial flutter 2/13 AF	AAD's/overdrive pacing /DCCV/ablation	Restoration of NSR = clinical improvement. Increased mortality in AF group
Zhang et al. (22)	Retrospective, 3-year, single center study	PAH Non- PAH PAF	PAH 100 Non-PAH 200	F:M 54.4:45.6% Age 62.9 + 6.8 MeanPAP 31.9+-6.2	X	Holter post ablation + 24-h Holter 3 monthly	11.3% early recurrence of PAF 7.3% Late recurrence of PAF	Attempted in all	All patients underwent radiofrequency ablation AAD's stopped 2 months post ablation	Suggests raised PAP increases chance of late recurrence PAF.

In patients with idiopathic PAH on disease specific therapies Wen et al. (19) undertook a prospective, cohort study with 280 patients recruited specifically with NSR at baseline. Forty patients developed SVA at least once during follow-up, 5-year incidence was calculated as 15.8%, potentially a more realistic reflection in PAH alone. Patients as with previous studies did not tolerate SVA and restoration of NSR correlated with clinical recovery. Studies again only diagnosed SVA on 12-lead ECG, potentially failing to diagnose asymptomatic SVA.

##### Treatment

AAD's, overdrive pacing, DCCV, and radiofrequency ablation are all guideline based therapies for SVA (6, 7), (although not PAH-specific). Existing data showed that rhythm control has been the most popular first line strategy and restoration of NSR demonstrated clinical improvement. Cannillo et al. (18) attempted rhythm control in 76% of patients diagnosed with SVA, NSR was restored in 11 (65%) cases but there was a high recurrence rate (~80%). Olsson et al. (20) diagnosed patients with SVA on 12-lead ECG and largely from patients presenting with deterioration of PAH/RHF symptoms rather than symptoms of arrhythmia [as per Cannillo et al. (18)]. Those with atrial flutter received ablation earlier with the concurrent use of AAD's with or without synchronized DCCV. For stable AF 10–14 days of oral amiodarone was prescribed pre DCCV and continued thereafter. If there were signs of RHF, amiodarone was given intravenously, and DCCV was performed. NSR was restored in 21/24 patients with atrial flutter and 16/24 patients with AF, resulting in clinical improvement. As such this observational study demonstrates that amiodarone was well-tolerated in PAH. Five-year survival in PAH/inoperable CTEPH was 68% with a fall to 58% if the patient developed a transient SVA and reduced further to 47% in permanent SVA. They also found that AF was more resistant to treatment than atrial flutter (similar to non-PAH disease). Consistent with these findings, Ruiz-Cano et al. (16) found that after the first episode of SVA (despite restoration of NSR/adequate rate control) 46.4% patients required an increase in PAH-specific medications secondary to progressive RHF, an interesting observation but not guideline directed. This data may suggest that the onset of SVA can be a prelude to RHF and/or clinical deterioration and that restoration of NSR or escalating PAH-specific therapy should be considered. Zhang et al. (22) specifically reviewed the efficacy and safety of electrophysiology studies and ablation in SVA and PAH. A retrospective study it reviewed 300 patients over 3 years [PAH 100 (observation) and non-PAH 200 (control)] with PAF undergoing ablation for the first time. All patients had a 24 h Holter monitor fitted immediately post ablation and AAD's were stopped 2 months later. EPS and ablation were found to be safe and reasonably effective in this cohort of patients. Bandorski et al. (23) agreed and found that 12/14 patients (all PH types) with a diagnosis of atrial flutter during EPS subsequently had successful ablation. Zhang et al. (22) noted that 11.3% of patients had an early recurrence PAF and 7.3% a late recurrence, with some correlation between a higher RAP and the incidence of late PAF recurrence.

In conclusion, incidence of SVA in PH (including PAH) ranges from 13.2 to 25.1% with a relationship to clinical worsening. As such this may be an indicator for restoration of NSR or alteration of PAH specific therapy. A range of therapeutic strategies have been investigated, however conclusions on efficacy are challenging to substantiate as the majority of studies were single arm. Little evidence of harm was identified. As in left heart disease NSR was easier to restore in atrial flutter vs. AF and all studies suggested that restoration of NSR resulted in improved clinical outcomes.

#### Ventricular Arrhythmias

The impact of ventricular arrhythmias in patients with PH is examined in a series of papers by Bandorski et al. An initial retrospective, two-center study (23) analyzed data from 55 PH patients presenting with indications for EPS (14 with group I PH). Fifteen had non-sustained ventricular tachycardia (NSVT) on a Holter monitor, however the prognostic relevance is unclear although likely to infer increased risk and extrapolation of prevalence from such a study is challenging due to small numbers and the large contribution of patients (23 in this study) with left heart disease-associated PH. There were no evidence suggesting EPS or ablation to be unsafe or ineffective in PH patients.

In a larger study, Bandorski et al. (24) sought to determine the incidence of VA in PH. Ninety two patients were enrolled in total (54 Group I, 10 Group 3, 26 Group 4, 2 Group 5), all of whom were on PAH-specific medication and in NSR at the time of enrolment. During 72-h Holter monitoring, 17 patients (18.5%) had a detectable arrhythmia [NSVT (12 patients), second degree atrioventricular block (1), intermittent complete heart block (1), and atrial flutter (1)]. Although small and including patients with all PH types, they undertook Holter monitoring and found arrhythmia in asymptomatic patients highlighting the potential inaccuracy of determining the incidence or prevalence of asymptomatic arrhythmia from 12-lead ECG. It highlighted that the use of Holter monitoring in this cohort was beneficial for arrhythmia diagnosis and this may be a useful tool in PAH patients also. To determine the prognostic significance of NSVT Bandorski et al. (25) examined 78 patients with PAH or inoperable CTEPH. Fifty-Five patients with PAH and 23 CTEPH underwent a clinical review, bloods, Holter monitoring, 6 MWT, echocardiography and RHC (25), of whom 12 had newly detected NSVT.

Relatively little evidence exists defining the prevalence and incidence of VA in PAH. Prevalence is estimated to be ~27% in all PH types with a considerable proportion of whom have co-existing left heart disease. Longer studies utilizing Holter monitoring rather than 12-lead ECG show higher rates of VA suggesting a bias in methods. The most reliable incidence of VA in all PH was ~15–18.5% based on Holter monitoring (25) suggesting a significant burden. Therapeutic options are unclear with only single arm studies available.

#### Bradyarrhythmia's

Limited data is available. Whilst determining the incidence of VA in PH Bandorski et al. (24), identified that 4/17 patients with newly diagnosed arrhythmias had intermittent heart block. Two patients, despite not taking rate limiting medication, progressed to complete heart block, and required pacemaker implantation. Studies are small and the relationship to poor outcome is uncertain.

#### Mode of Death

The predominant cause of death in patients with PAH is thought to be RHF or sudden cardiac death (SCD) (26). Hoeper et al. (27) undertook a retrospective, multi-center (17 referral centers in Europe and the United States) looking at patients with PAH who had developed cardiac arrest. 3130 patients with PAH were treated over 3 years and 513 had circulatory arrest. Cardiopulmonary resuscitation (CPR) was attempted in 132 (26%) but despite the majority occurring in a hospital setting only 8 patients (6%) survived to > 90 days. No apparent differences were found preceding cardiac arrest accounting for why a patient did or did not survive. It is difficult to fully assess cause of death, this data showed that 54% of patients were admitted with intercurrent illness; 49% died from progressive RHF; 18% respiratory failure, and 8% from other causes. Seventeen percent died from SCD and it is unclear as to whether this was secondary to PAH progression or potentially treatable arrhythmia.

## Conclusion

Present data suggest that patients with PH have increased risk of arrhythmia, however accurate estimates of incidence and prevalence in patients with PAH remain elusive. PAH is a rare disease and as such patient numbers are often limited and the studies undertaken have grouped patients with PAH with other forms of related disease. As such, longitudinal studies with defined enrolment criteria are required to determine the arrhythmic burden of patients with PAH. The enrolment of patients with pre-existing symptoms is a potential source of bias exemplified by the increased rates of asymptomatic SVA and VA (24) identified with prolonged monitoring. Arrhythmias have been demonstrated to precede adverse clinical events and it is therefore of clinical importance to accurately define prevalence and incidence and examine potential therapeutic options. Current evidence highlights gaps in our knowledge as we only have “snapshots” of data from a 12-Lead ECG or Holter monitoring. Advances in technology now allow for long term monitoring of cardiac rhythm and as such a prospective study with continuous monitoring may further inform incidence, prevalence and relationships to adverse outcomes, prior to studies of therapeutic strategies.

## Author Contributions

JM and AR wrote the manuscript draft. All authors critically reviewed the paper and approved the final manuscript for submission.

### Conflict of Interest Statement

The authors declare that the research was conducted in the absence of any commercial or financial relationships that could be construed as a potential conflict of interest.

## Nomenclature

6MWT: 6-minute walk test
AAD: Antiarrhythmic drugs
AF: Atrial fibrillation
AVNRT: Atrioventricular node re-entry tachycardia
BP: Blood pressure
BNP: Brain natriuretic protein
CO: Cardiac output
COPD: Chronic obstructive pulmonary disease
CPR: Cardiopulmonary resuscitation
CTEPH: Chronic thromboembolic pulmonary hypertension
DCCV: Direct current cardioversion
ECG: Electrocardiogram
EPS: Electrophysiology study
LVEF: Left ventricular ejection fraction
NSR: Normal sinus rhythm
NSVT: Non-sustained ventricular tachycardia
PAF: Paroxysmal atrial fibrillation
PAH: Pulmonary arterial hypertension
PAP: Pulmonary arterial pressure
PCWP: Pulmonary capillary wedge pressure
PH: Pulmonary hypertension
PVR: Pulmonary vascular resistance
RAP: Right atrial pressure
RHC: Right heart catheter
RHF: Right heart failure
SCD: Sudden cardiac death
SSc-PH: Systemic sclerosis-associated pulmonary hypertension
SVA: Supraventricular arrhythmia
VA: Ventricular arrhythmia
WHO: World health organization
